# C-reactive protein in a naturalistic sample of inpatients with major depressive disorder, bipolar disorder and obsessive-compulsive disorder

**DOI:** 10.1192/j.eurpsy.2021.2019

**Published:** 2021-08-13

**Authors:** D. Caldirola, S. Daccò, F. Cuniberti, G. Diaferia, P. Cavedini, M. Grassi, G. Perna

**Affiliations:** 1 Mental Health, Humanitas University, Rozzano, Italy; 2 Department Of Clinical Neurosciences, Villa San Benedetto Menni Hospital, Hermanas Hospitalarias, Albese con Cassano, Italy

**Keywords:** major depressive disorder, bipolar disorder, Obsessive-Compulsive disorder, Inflammation

## Abstract

**Introduction:**

The relevance of inflammation to psychiatric disorders is well established. While inflammation was broadly investigated in mood disorders, obsessive-compulsive disorder (OCD) received little attention. C-reactive protein (CRP) is an inflammatory marker commonly assessed in clinical practice. Studies comparing CRP levels across mood disorders and OCD are lacking.

**Objectives:**

We compared the prevalence of CRP-based low-grade inflammation (LGI) across major depressive disorder (MDD), bipolar disorder (BD) and OCD, taking into account multiple individual variables that may affect CRP levels.

**Methods:**

Retrospective, observational cross-sectional study in a naturalistic sample of hospitalized patients with MDD or BD or OCD. Information was collected from electronic medical records. Based on serum CRP levels at admission, the following were defined: CRP: >3 mg/L and ≤10 mg/l, “yes” LGI; ≤3 mg/L, “no” LGI. Logistic regression models were applied.

**Results:**

We included 156 patients with MDD, 135 with BD, and 97 with OCD. We found prevalence rates of CRP-LGI of 29.9%, 36.5%, and 47.4% in patients with OCD, MDD, and BD, respectively, without significant differences between groups. The entire set of individual variables considered (e.g., sex, body mass index, medication) explained only one-third of the observed variations in CRP-LGI.

**Conclusions:**

CRP-LGI may be a transdiagnostic feature of a substantial portion of patients with MDD or BD or OCD, rather than being exclusive to a specific psychiatric disorder. The presence of LGI was not fully explained by individual confounding factors. Given the relevance of inflammation to psychiatric and medical outcome, routine measurements of CRP in psychiatric settings may be valuable.

**Disclosure:**

No significant relationships.

